# Predictors of pre-hospital vs. hospital mortality due to road traffic injuries in an Iranian population: results from Tabriz integrated road traffic injury registry

**DOI:** 10.1186/s12873-022-00593-w

**Published:** 2022-03-08

**Authors:** Sadeghi-Bazargani Homayoun, Jamali-Dolatabad Milad, Golestani Mina, Sarbakhsh Parvin

**Affiliations:** 1 Research Center for Evidence-Based Medicine, Faculty of Medicine, Tabriz University of Medical Sciences, Tabriz, Iran; 2 Road Traffic Injury Research Center, Tabriz University of Medical Sciences, Tabriz, Iran; 3 Department of Statistics and Epidemiology School of Public Health, Tabriz University of Medical Sciences, Tabriz, Iran

**Keywords:** Road traffic, Accidents, Brain trauma, Mortality, Hospital death, Pre-hospital

## Abstract

**Background:**

Road Traffic Injuries (RTIs) is considered as one of the main health challenges and causes of mortality, worldwide and especially in Iran. Predicting the place where RTIs-related death takes place is vital in decreasing this type of mortality. The purpose of the present study was to identify the predictors of RTI fatalities with respect to the place of death (hospital vs. pre-hospital) during the recent decade in East Azerbaijan Province, Iran.

**Methods:**

Overall, 7347 RTI fatalities were retrieved from the road traffic injuries registry which is supported by the Forensic Medicine Organization in East Azerbaijan. Among these cases, 2758(37.5%)) were hospital deaths. The registered variables of these cases were analysed using bivariate and multiple logistic regression (STATA version 15).

**Results:**

Out of 7347 deaths, 5862 (79.8%) were men and the rest were women 1485 (20.2%).The mean age was 40.3 (SD = 20.8). Of the total number of cases, 2758 (37.5%) died in hospital death and the rest 4589 (62.5) were pre-hospital death. According to the results of the present study, inter-city RTI (OR = 1.7, CI 95% = (1.5–2)) and RTIs inside the city of Tabriz (OR = 1.4, CI 95% = (1.2–1.6)) increases the chance of hospitals death. In addition, having a heavy counterpart vehicle compared to no counterpart vehicle decreased the chances of hospitals death (OR = 0.46, CI 95% = (0.39–0.55)) while motorcycle or bike counterpart vehicle compared to no counterpart vehicle increased the chances of hospital death (OR = 2.26, CI 95% = (1.59–3.22)). Also the users of the motorcycle or bike vehicle compared to the pedestrians increased the chances of hospital death (OR = 1.43, CI 95% = (1.19–1.71)) while any the other vehicle users compared to the pedestrians have significantly lower chances for hospital death. Other factors that increased hospitals death were transferring injured people by ambulance (OR = 1.3, CI 95% = (1.1–1.6)) and being elderly (OR = 1.5, CI 95% = (1.2–1.7)). Moreover, it was found that the annual trend of change in hospital death is strongly affected by the above-identified factors.

**Conclusions:**

The effective predictors in hospital death were RTI location, type of counterpart vehicle, used vehicles and lighting condition. The identified factors related to the location of deaths by RTI can be divided into the RTI severity-related factors as well as factors related to the services quality and speed of delivery. According to the present results, through professional training of people in the field and providing immediate assistance in RTIs pre-hospital mortality can be significantly prevented.

## Background

Today, Road Traffic Injuries (RTIs) is considered as a novel public health challenge across the globe [1]. Annually, at the global level, over 1.35 million people are killed and roughly, 50 million people are left injured due to RTIs. Most of these events happen in Low and Middle Income Countries (LMICs). RTIs are the 8th cause of death, worldwide and the 1st cause of mortality in 15–29 age group [2]. It is estimated that in case of the absence of effective actions, until 2020, RTIs-related mortality in LMICs will increase by 80% [3]. According to the estimation by the World Health Organization (WHO) in Iran, RTAs-related death rate is 20.5 per100 000 people which makes RTAs as Iran’s 5th leading cause of death and largest cause of Years of Life Lost (YLL) [2]. This challenge increases both direct expenses including treatment expenses and provision of care for the injured cases in accidents and indirect expenses such as psychological problems and depression in family members and temporal or permanent loss of active work force [4]. RTIs-related deaths may occur at the scene of the accidents, on the way to the hospital or at the hospital. To reduce mortality, it is essential to predict where death will occur. One of the effective ways of immediate prevention RTI-related mortality is the provision of timely and instant help [5]. The place where death occurs could be linked to different factors. Identification of these factors is vitally important in decreasing mortality through planning and intervention. According to the global studies, at least 39% of mortality arising from RTIs happen before they reach to the hospitals. In fact, through effective planning and interventional programs, RTIs mortality can be significantly reduced [5–7]. Many RTIs studies, particularly descriptive cases, have been carried out in Iran, so far. However, there is not enough studies on RTIs-caused death place either at the hospital and deaths before arriving at hospital [8, 9]. More importantly, in carried out studies less attention has been paid on the predictors of pre-hospital and hospital deaths. The present investigation is a part of a project study on RTIs-related mortality and injuries [10, 11]. It aimed to study the trend of RTIs mortality and identify the predictors of hospital death in East Azerbaijan province to address the factors affecting deaths incidence. It has been said that RTIs-related hospital deaths happen due to the lower hospital services and therefore, in the present study it has been tried to evaluate the changes in terms of hospital deaths and affecting factors on the rate of deaths related to the place of death. Through the evaluation, we found that a small number of studies has been carried out on the subject matter. Identifying these factors play key role in policy making to decrease mortality as well as using evidence-based decisions based on the importance of factors involved in death incidence.

## Methods

The present study was conducted utilizing data taken from the registry of road traffic injuries supported by the Forensic Medicine Organization of East-Azerbaijan province, Iran in March 2010–March 2019. According to the national census in 2011, East-Azerbaijan province, located in the northeast of the country, had a population of 3,725,000. In this study, the entire mortality registered between March 2010 and March 2019 was studied. The predictors of hospital death were also considered in these dead people. The data of the current study, collected by Forensic Medicine Organization, covers the whole province and according to the WHO definition, the deaths occurred until thirty days after RTIs is considered as traffic death. All 7785 cases, registered in March 2010–March 2019 in Forensic Medicine Organization, were evaluated. Out of the total number, 377 (4.8%) cases were excluded because either they happened outside the province or they died 30 days after the accident. Finally, 7408 matched WHO definition and were eligible to be included. Due to the insufficient data, 61 (0.8%) cases were excluded from final analysis and remaining 7347 cases were included. The utilized data included demographic variables (age, gender and education), accidents mechanism, accidents places and conditions, specifications of the vehicles used (used by the deceased) and counterpart vehicle (the vehicle that has collided with itself or the vehicle of a deceased person) in the accidents, data on the damages happened to the injured people and their details, and data collection tools being published as a research protocol in the study [10]. Microsoft Access (version 2016) was used to prepare the data for preliminary analysis. Then, they were fed into STATA (version 15) for further analysis. First, descriptive statistics such as frequency mean and Standard Deviation (SD) were used. Then, considering hospital death as the response variable, and in order to identify predictors of hospital death, initially the variables were analyzed in the form of bivariate (chi-square test for categorical scale variables and t-test for quantitative variables). Next, the variables that were significant in bivariate test at the 0.1 level were included in the multiple logistic analysis. The modeling process was based on the stepwise methods. The missing values were handled using “listwise deletion “, meaning that any observations that are not in the outcome variable or any of the prediction variables were deleted.

Integrated Road Traffic Injuries Registry (IRTIR) has been approved by the Road Traffic Injury Research Center under number 700/1482 and jointly supported by Iranian Ministry of Health under contract number 700/D/581 and by WHO under contract number 2017/742294–0. It has received ethical approval under number IR.TBZMED.REC.1396.465 from the Ethical Committee in Tabriz University of Medical Sciences.

## Results

Out of 7347 deaths, 5862 (79.8%) were male and the remaining 1485 (20.2%) were female. The mean age was 40.3 (SD = 20.8). Hospital deaths were 2758(37.5%) and the rest 4589 (62.5%) were pre-hospital death.

**Fig. 1:**
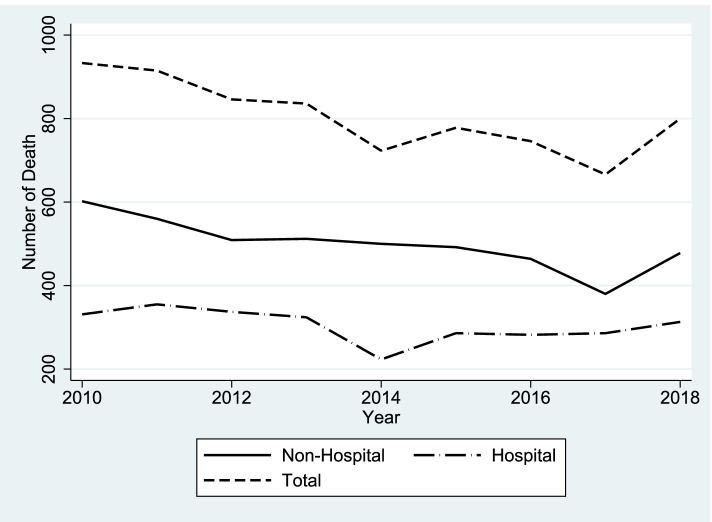
The trend of hospital and pre-hospital death incidence caused by RTIs in East Azerbaijan during 2010 and 2018

Figure 1 displays the total mortality of hospital and pre-hospital death for a period of 9 years. As illustrated in the Fig. 1, in general, the total RTIs-related mortality is decreased. According to the Fig. 1, it can be seen that pre-hospital death has a slope almost similar to the total number of deaths, but this slope is almost constant for hospital death that after 2014, this slope is somewhat increasing.

Logistic regression method was used to identify the factors affecting hospital and pre-hospital death. To create a multiple logistic model, the variables were first analysed as bivariate and those that were significant at the 0.1 level were entered into the modelling which was done taking into account the importance of the variables. To enter the model, there was a significant relationship with death in the hospital (*p* < 0.1). The mean age of hospital deaths was 42.7(SD = 22.3) and for pre -hospital cases it was 38.9 (SD = 19.7) that was statistically significant (*P*-value< 0.001). The results of categorical scale variables are shown in Table 1. The difference in the distribution of variables has been evaluated using chi-square test.

**Table 1:** Frequency distribution of road traffic fatalities in East Azerbaijan 2010–2018

Variable	Attributes	Hospital death N (%)	Pre-hospital death	*P*-value	Missing N (%)
Gender	Male	2237(38.16%)	3625(61.84%)	0.029	0
	Female	521(35.08%)	964(64.92%)		
Education	Illiterate	774(41.28%)	1101(58.72%)	0.001	177(2.41%)
	Diploma	1408(37.66%)	2331(62.34%)		
	Graduate	548(35.22%)	1008(64.78%)		
The role of deceased	Driver/rider	1073(35.77%)	1927(64.23%)	< 0.001	65(0.88%)
	Pedestrian	886(50.17%)	880(49.83%)		
	Passenger or a pillion passenger	783(31.12%)	1733(68.88%)		
Place of accident	Inner city	1083(56.23%)	843(43.77%)	< 0.001	56(076%)
	Sub-urban	1646(30.68%)	3719(69.32%)		
Accident district	Tabriz city	886(51.30%)	841(48.70%)	< 0.001	1(0.01)
	Others	1872(33.32%)	3747(66.68%)		
Vehicle used	Light vehicles	1066(28.42%)	2685(71.58%)	< 0.001	85(1.16%)
	Pedestrian	881(50.29%)	871(49.71%)		
	Heavy vehicles	71(22.68%)	242(77.32%)		
	Motorcycle & bike	690(52.23%)	631(47.77%)		
	Other	21(16.80%)	104(83.20%)		
Counterpart vehicle	Light vehicles	1367(44.15%)	1729(55.85%)	< 0.001	342(4.65%)
	No counterpart vehicle	661(35.44%)	1204(64.56%)		
	Heavy vehicles	369(23.35%)	1211(76.65%)		
	Motorcycle & bike	161(71.11%)	65(28.89%)		
	Other	82(34.31%)	157(65.69%)		
Lighting status	Day light	1742(37.92%)	2852(62.08%)	0.011	405(5.51%)
	Night	596(34.25%)	1144(65.75%)		
	Twilight	208(34.21%)	400(65.79%)		
Injured transport vehicle	Ambulance	2284(37.73%)	3770(62.27%)	0.040	608(8.28%)
	Other	231(33.72%)	454(66.28%)		
Injured organs	Head and face	2124(35.48%)	3863(64.52%)	< 0.001	0
	Other	634(46.62%)	726(53.38%)		
Cause of death	Head trauma	1587(36.52%)	2758(63.48%)	< 0.001	12(0.16%)
	Bleeding	243(43.86%)	311(56.14%)		
	Multiple fractures	435(39.62%)	663(60.38%)		
	Burns and choking	18(16.22%)	93(83.78%)		
	Mixed causes	413(36.74%)	711(63.26%)		
	Other	57(55.34%)	46(44.66%)		
Accident mechanism	Vehicle-Vehicle crash	1115(32.70%)	2295(67.30%)	< 0.001	136(1.85%)
	Vehicle-pedestrian	887(50.46%)	871(49.54%)		
	Rollover	471(33.81%)	922(66.19%)		
	Other	237(36.46%)	413(63.54%)		

In the final multiple logistic analysis, variables with a significant relationship in the previous stage entered the model. The results of multiple logistic analysis can be seen in Table 2.

**Table 2:** Multiple Logistic Analysis factors of hospital death caused by traffic accidents in East Azerbaijan 2010–2018

Variable	Level of the variables	Or	*P*-value	95% CI	
Place of accident	Sub-urban	Ref. Group			
	Inner city	1.69	< 0.001	1.46	1.97
Accident district	Tabriz city	Ref. Group			
	Others	1.37	< 0.001	1.18	1.60
Counterpart vehicle	No counterpart vehicle	Ref. Group			
	Light vehicles	0.87	0.084	0.75	1.02
	Heavy vehicles	0.46	< 0.001	0.39	0.55
	Motorcycle & bike	2.26	< 0.001	1.59	3.22
	Other	0.69	0.033	0.49	0.97
Vehicle used	No vehicle used	Ref. Group			
	Light vehicles	0.63	< 0.001	0.53	0.75
	Heavy vehicles	0.52	< 0.001	0.37	0.73
	Motorcycle & bikes	1.43	< 0.001	1.19	1.71
	Other	0.33	< 0.001	0.19	0.57
Injured transport vehicle	Other	Ref. Group			
	Ambulance	1.34	0.003	1.10	1.61
Damaged parts	Other	Ref. Group			
	Head and face	0.56	< 0.001	0.47	0.67
Cause of death	Head trauma	Ref. Group			
	Bleeding	1.14	0.288	0.90	1.44
	Multiple fractures	0.97	0.771	0.81	1.17
	Burns and choking	0.26	< 0.001	0.14	0.49
	Mixed causes	0.90	0.245	0.76	1.07
	Other	1.57	0.065	0.97	2.52
Lighting status	Day light	Ref. Group			
	Night	0.77	< 0.001	0.68	0.88
	Twilight	0.87	0.177	0.71	1.06
Elderly status	Age < 65	Ref. Group			
	Age > 65	1.47	< 0.001	1.24	1.73

**Fig. 2:**
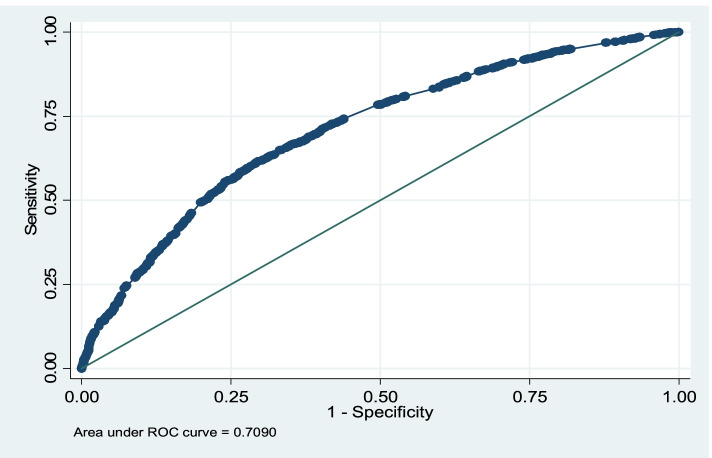
ROC diagram of fitted model for hospital and pre-hospital death incidence caused by RTIs in East Azerbaijan during 2010 and 2018

The variables yielded for predicting RTI-related hospital death, after controlling for other variables and multiple logistic analysis, are presented in details in Table 2. The odd of hospital death occurred in Tabriz was 37% higher when compared to other cities (OR = 1.37, *P*-value< 0.001) and the hospital death occurred in intra-city was 69% higher compared to suburban (OR = 1.69, *P*-value< 0.001). According to the Table 2, heavy counterpart vehicle and other vehicles (road maintenance vehicle, agricultural and military vehicles, etc.) compared to no counterpart vehicle, decreased the hospitals death odd, respectively as 44 and 31%. It is while, motorcycle and bicycle, as counterpart vehicle increased hospital death up to 2.26 times when compared to no counterpart vehicle. In cases in which the used vehicles were light vehicles, trucks, and others (road maintenance, agricultural, military, etc.), hospital deaths were less than pedestrians. In cases that crashed with motorcycle or bike, hospital death was higher when compared to pedestrians. In those cases which were carried by ambulance, hospital death was 34% higher than other ways of transfer (OR = 1.34, *P*-value =0.003). Compared to other parts of the body, hospital death among cases who injured by their head or neck was 44% lower (OR = 0.56, *P*-value < 0.001). The cases with suffocation or burn as their death reason had 74% decreased hospital death when compared to head trauma (OR = 0.26, *P*-value < 0.001). Died cases who were injured at night had 23% probability of hospital death when compared to those in day (OR = 0.77, *P*-value = 0.001). Finally, injured people above 65 years had 1.47 times higher probability of hospital death compared to younger cases (OR = 1.47, *P*-value < 0.001). The final fitted model (Likelihood-ratio test) was significantly reliable (chi2 = 780.23, df = 20, *P*-value< 0.001). Figure 2 shows the ROC curve for the fitted model. Area under the ROC curve (AUC = 0.71) and correct classification rate (CCR = 68.17%), suggest relatively good performance of fitted model in predicting hospital death.

## Discussion

The present study tries to identify the predictors of RTIs-related pre-hospital and hospital death in East Azerbaijan Province, Iran. It shows that the RTIs-related hospital death has been decreased during a period of 9 years. Moreover, despite decrease in total deaths, hospital deaths had fixed trend and even increased slightly, but we can see the decrease in pre-hospital deaths. The evaluation of hospital death predictors should focus on the less severity of inner-city RTIs when compared to sub-urban cases. In addition, it can be due to the heaviness of sub-urban vehicles. In other words, it can be said that most sub-urban cases usually die before they arrive at the hospital. Another factor is the immediate medical aid inside the city (especially the city of Tabriz as the capital of province). In most of the studies, it has been reported that the fatality of sub-urban RTIs was higher than inner-city cases [12, 13]. The studies also showed that pre-hospital death was very higher in rural areas [14, 15]. The results of the study suggested that motorcycle and bikes as counterpart or used vehicle in RTIs, can increase hospital death when compared to the situation in which they are absent in RTIs. Studies about the predictors of pre-hospital death showed the similar results to that of the present investigation [13, 16–18]. Hospital death in cases who were carried by ambulance to the hospital was higher than those cases carried by other vehicles. It means faster and specialized aid provided to RTI injured people. In other words, ambulance delays death. Many studies have shown that the quality of services can play role in early death [19–21]. Hospital death in cases with head and face injuries was higher. It can be due to the lower survival of these people who face with head trauma. This finding was in agreement with the results of other studies on the RTIs-related injuries of different parts of the body [22, 23]. In other words, people who had injured by their face and head did not have enough time to be transferred to the hospital. In addition, those people with suffocation or burn as their death reason have lower hospital death when compared to people with head trauma. This can be due to the quick death of these cases before they are transfer to the hospital. It can be said that the severity of trauma is so high that they loss the chance of arriving to hospital. This finding was in agreement with other studies [16–22, 24]. RTIs at night had higher hospital death when compared to the RTIs happen in the morning. Due to the uncrowdedness at nights drivers are less tended to follow the rules such as drinking alcohol or using drugs or crossing red light. On the other hand, poor lighting and sleepy drivers can increase RTA severity and death at the scene of accident. This findings was also in agreement with the findings of other investigations [17, 18]. Hospital death among people above 65 was higher, probably because most of these accidents happened inside the city. Another explanation is that the most of the elderly who lose their lives, due to common and chronic diseases, die from the consequences of trauma even it would be less severe. This finding was in agreement with that of the other studies [11, 12, 14–16, 25]. However, it was not in agreement with the study carried out in Japan. The Japanese authors found that elderly above 65 had higher pre-hospital death. This can be due the difference in the studied population because they only studied inner-city RTIs [17]. High-quality Emergency Medical Services (EMS) provided through emergency cares at the RTIs scene and medical care during rapid transfer of victims to hospitals are significantly important in reducing mortality [26, 27]. In 2009, Iran’s Ministry of Health and Medical Education improved the quality of its EMS. Seven times increase in the number of the ambulance, four times increase in EMS posts and significant reduction in transfer time should not be neglected when studying the reasons of reduction in mortality rate in recent years [28].

### Limitations

Despite the development of data collection tool by national experts and its assessment in the Forensic Medicine Organization, the validity and confidence level of the tools has not been published so far, but the recently published research protocol provides more details on the research methodology [10]. Data collection tools may affect the quality of the output. Also, due to the legal limitations in the country, death during transfer due to RTIs as well as deaths at the scene of accident have no meaning and are called hospital death. Also, because the present data were taken from the registry system and because it used the Minimum Data Set (MDS), in this study to the authors evaluated the factors used in MDS.

## Conclusion

Effective predictors in hospital death were RTI location, the type of counterpart vehicle and used vehicle, and lighting condition. The identified factors related to the location of deaths due to RTI can be divided into to the RTI severity-related factors as well as factors related to the quality and speed of delivery services. Based on the results of the present study, providing immediate assistance in RTIs can significantly prevent from pre-hospital mortality. By professional training of people in the field of provision of aid in RTIs the speed of aid and immediate transfer of the injured to the hospitals can be promoted and improved. Furthermore, with the presence of specialty hospitals in the field of RTIs and allocation of efficient therapeutic facilities in these hospitals, traffic mortality can be greatly decreased.

## Data Availability

The datasets analysed during the current study are not publicly available because the utilized data were registered in the Forensic Medicine Organization and they do not belong to the researchers of this study but are available from the corresponding author on reasonable request.

## Abbreviations

RTIs: Road Traffic Injuries
LMICs: Low and Middle Income Countries
WHO: World Health Organization
SD: Standard Deviation
OR: Odds Ratio
ROC: Receiver Operating Characteristic.
AUC: Area under the ROC Curve
CCR: Correct Classification Rate
EMS: Emergency Medical Services
IRTIR: Integrated Road Traffic Injuries Registry
